# On Compressing the Femoral Artery at the Groin

**Published:** 1806-07

**Authors:** John Ring

**Affiliations:** Member of the Royal College of Surgeons, in London


*On compressing the Femoral Artery at the Groin.* By Mr. JOHN RING, Jun. *Member of the Royal College of Surgeons, in London.*

I Am induced to offer you the following case for insertion in your Journal, as affording a strong illustration of two points of practical importance:

1st. That the femoral artery may be successfully compressed at the groin, even under the most disadvantageous circumstances.

2dly.

2dly. That the operation of tying this artery will sometimes succeed under appearances the most discouraging.

J. Hiscock, of Sturminster Newton, Dorset, a shoemaker, in the act of cutting a piece of leather with a long slender knife, wounded the femoral artery; a sudden gush of blood took place, followed by a swelling of the wounded part, and syncope. In this state he was put to bed. For some days he got up occasionally, using discutient applications; the real nature of the wound not being ascertained. The swelling, however, continuing to increase, attended with a pulsation, removed all doubts respecting it. At the expiration of three weeks, I was consulted, and found him reduced by repeated hæmorrhages; a flushed countenance, and a slight cough; the whole thigh swollen and inflamed, and the tumor on the inside of it, extending about nine inches, in the middle of which was the wound, now enlarged to nearly the size of a shilling, stopped up by a coagulum. No time was to be lost. I recommended the operation, which was immediately assented to, and I was requested to perform it. Expecting considerable difficulty, I availed myself of the assistance of my friend Mr. Surrage of Wincanton, as well as that of the practitioner who consulted me. Having seen several instances of accidents occurring during the performance of operations, from the brass or tape of the tourniquet giving way, we procured a common sized door key, and filled the bowl of it with broad tape, so as to make a good compress.

After screwing the tourniquet as tight as we possibly could, I began my incision from the lower part of the tumor upwards, and had no sooner reached the wound, and slightly loosened the coagulum filling it, with the knife, than a sudden gush of blood took place from the artery. On Mr. Surrage's attempting to tighten the tourniquet, the tape snapped. I immediately pushed my hand through the coagula, to compress the artery against the bone, and Mr. Surrage applied the key to the groin. I soon found, by the pulsation of the artery ceasing, that the pressure at the groin was effectual; and on removing my fingers no hæmorrhage ensued. Fearing Mr. Surrage would not be able to keep an adequate pressure, during the operation, and as a comparatively small loss of blood would be attended with fatal consequences, a second tourniquet was applied, but, from the swollen and inflamed state of the thigh, it was found wholly insufficient to stop the circulation; and we were under the necessity of trusting to the compression on the groin. The incision, to the length of the tumor, being completed, compleated, and about three pounds of coagulated blood removed, the fascia, containing the vessels, was la d open; and the vein being drawn aside, an incised wound, about the third of an inch, was discovered in the artery. Not a drop of blood was flowing from it. A double ligature was then passed under the artery, the upper one being tied, and the pressure at the groin removed. In a few moments, a small stream issued from the lower extremity. The lower ligature was then tied, the artery divided between them, and both ligatures secured in the manner recommended by Mr. H. Cline. The sides of the cavity were supported by slips of adhesive plaster, the integuments not admitting of sutures. In a few days an extensive suppuration came on, attended with considerable sloughing, which gradually lessened, and in about a month entirely ceased. The lower ligature separated on the tenth day, and the upper one on the twenty-first. In six weeks the wound was healed. The patient at first walked on his toes, from the bent position of his leg during his long confinement. He now walks nearly as well as ever; and the thigh is recovering its natural size.

*South Molton Street.*

